# The contribution of pathogenic variants in breast cancer susceptibility genes to familial breast cancer risk

**DOI:** 10.1038/s41523-017-0024-8

**Published:** 2017-06-09

**Authors:** Thomas P. Slavin, Kara N. Maxwell, Jenna Lilyquist, Joseph Vijai, Susan L. Neuhausen, Steven N. Hart, Vignesh Ravichandran, Tinu Thomas, Ann Maria, Danylo Villano, Kasmintan A. Schrader, Raymond Moore, Chunling Hu, Bradley Wubbenhorst, Brandon M. Wenz, Kurt D’Andrea, Mark E. Robson, Paolo Peterlongo, Bernardo Bonanni, James M. Ford, Judy E. Garber, Susan M. Domchek, Csilla Szabo, Kenneth Offit, Katherine L. Nathanson, Jeffrey N. Weitzel, Fergus J. Couch

**Affiliations:** 1 Department of Medical Oncology, Division of Clinical Cancer Genetics, City of Hope, Duarte, CA USA; 2 Department of Population Sciences, Beckman Research Institute of City of Hope, Duarte, CA USA; 3 Department of Medicine, Division of Hematology-Oncology, Perelman School of Medicine University of Pennsylvania, Philadelphia, PA USA; 4 Abramson Cancer Center, Perelman School of Medicine at the University of Pennsylvania, Philadelphia, PA USA; 5 Department of Health Sciences Research, Mayo Clinic, Rochester, MN USA; 6 Department of Laboratory Medicine and Pathology, Mayo Clinic, Rochester, MN USA; 7 Clinical Genetics Research Lab, Department of Medicine & Department of Cancer Biology and Genetics, Memorial Sloan Kettering Cancer Center, New York, NY USA; 8 Department of Molecular Oncology, British Columbia Cancer Agency, Vancouver, BC Canada; 9 Department of Medical Genetics, British Columbia Cancer Agency, Vancouver, BC Canada; 10 Department of Medicine, Division of Translational Medicine and Genetics, Perelman School of Medicine at the University of Pennsylvania, Philadelphia, PA USA; 11 Clinical Genetics Service, Department of Medicine, Memorial Sloan Kettering Cancer Center, New York, NY USA; 12 IFOM, the FIRC Institute of Molecular Oncology, Milan, Italy; 13 Division of Cancer Prevention and Genetics, European Institute of Oncology, Milan, Italy; 14 Division of Oncology, Stanford University School of Medicine, Stanford, CA USA; 15 Center for Cancer Genetics and Prevention, Dana Farber Cancer Institute, Boston, MA USA; 16 National Institutes of Health, Bethesda, MD USA

## Abstract

Understanding the gene-specific risks for development of breast cancer will lead to improved clinical care for those carrying germline mutations in cancer predisposition genes. We sought to detail the spectrum of mutations and refine risk estimates for known and proposed breast cancer susceptibility genes. Targeted massively-parallel sequencing was performed to identify mutations and copy number variants in 26 known or proposed breast cancer susceptibility genes in 2134 *BRCA1/2-*negative women with familial breast cancer (proband with breast cancer and a family history of breast or ovarian cancer) from a largely European–Caucasian multi-institutional cohort. Case–control analysis was performed comparing the frequency of internally classified mutations identified in familial breast cancer women to Exome Aggregation Consortium controls. Mutations were identified in 8.2% of familial breast cancer women, including mutations in high-risk (odds ratio > 5) (1.4%) and moderate-risk genes (2 < odds ratio < 5) (2.9%). The remaining familial breast cancer women had mutations in proposed breast cancer genes (1.7%), Lynch syndrome genes (0.5%), and six cases had two mutations (0.3%). Case–control analysis demonstrated associations with familial breast cancer for *ATM, PALB2*, and *TP53* mutations (odds ratio > 3.0, *p* < 10^−4^), *BARD1* mutations (odds ratio = 3.2, *p* = 0.012), and *CHEK2* truncating mutations (odds ratio* = *1.6, *p* = 0.041). Our results demonstrate that approximately 4.7% of *BRCA1/2* negative familial breast cancer women have mutations in genes statistically associated with breast cancer. We classified *PALB2* and *TP53* as high-risk, *ATM* and *BARD1* as moderate risk, and *CHEK2* truncating mutations as low risk breast cancer predisposition genes. This study demonstrates that large case–control studies are needed to fully evaluate the breast cancer risks associated with mutations in moderate-risk and proposed susceptibility genes.

## Familial breast cancer: Pinning down susceptibility genes beyond BRCA

Women with the heritable form of breast cancer often harbor mutations in cancer-linked genes other than the usual suspects, *BRCA1* and *BRCA2*. Slavin, Maxwell, Lilyquist, Joseph, and colleagues from major national and international cancer centers studied 2134 women with familial breast cancer who tested negative for *BRCA1/2* gene mutations. The researchers sequenced 26 known or proposed breast cancer susceptibility genes and found mutations in approximately 1 in every 12 of the study subjects. They then further broke down the susceptibility genes into those that confer high-, moderate- or low-risk—although not all the proposed breast cancer genes reached statistical significance and, as such, their clinical importance remains unclear. The results support adding some of the high- and moderate-risk genes to multi-panel diagnostic tests that aim to determine the likelihood of a women developing heritable breast cancer.

## Introduction

Breast cancer is the leading cause of cancer in women in the United States and the third leading cause of cancer death.^1^ Morbidity and mortality can be reduced by identification and clinical management of patients at high-risk.^2^ One major factor underlying breast cancer risk is genetic predisposition. It is currently thought that up to 10% of all breast cancers are due to an autosomal dominant susceptibility allele.^3, 4^ Mutant alleles in *BRCA1* or *BRCA2 (BRCA)* account for the majority of hereditary breast cancer susceptibility.^5^ However, many other risk-associated alleles in other genes have now been identified through the use of multigene panels in clinical and research applications.^6^ An improved understanding of the spectrum of genetic susceptibility and clarification of gene-specific risks for breast cancer may result in enhanced screening, prevention, and therapeutic strategies for patients and their families.^2, 4^

There are few studies focusing on mutations in breast cancer susceptibility genes in affected individuals who have two or more close relatives with breast or ovarian cancer but are BRCA1/2 mutation negative.^7–14^ The purpose of this study was to detail the spectrum of known and proposed breast cancer susceptibility genes, highlight genotype–phenotype correlations, and refine risk estimates for mutations in these genes by examining *BRCA* negative familial breast cancer (FBC; proband with breast cancer and a family history of breast or ovarian cancer; see methods) women compared to the non-Finnish European controls from the Exome Aggregation Consortium (ExAC).^15^

## Results

Phenotypic information and an overview of family history information for the 2134 individuals in the high-risk FBC is provided in Table 1. Participants were enrolled from multiple centers (see Methods). There were 2425 breast cancers in the 2134 women, with two breast cancers reported in 14% of these women. Full pathological information was available for 1055 tumors, and 62% of tumors were ER+HER2−, 16% were ER+HER2+, 9% ER−HER2+, and 14% were ER−HER2−. The majority of the cases (81%) were of European–Caucasian descent (Table 1). All FBC women had a family history of cancer, and 65% of women had at least one first-degree relative with breast cancer. A family history of ovarian cancer in at least one first or second degree relative was reported for 18% of women.

**Table 1:** Phenotypic and pathological features of 2134 women with familial breast cancer

	*n*	%
Race^a^		
White	1722	80.7
Hispanic	136	6.4
Asian	69	3.2
Other	69	3.2
African American	49	2.3
Unknown	89	4.2
Personal history of cancer^b^		
Second breast cancer	291	13.6
Ovarian cancer	33	1.5
Other cancer	167	7.8
Avg age 1st breast cancer	47.9 ± 9.4	
Avg age 2nd breast cancer	52.9 ± 10.5	
Family history of cancer		
No FDR/SDR w/breast cancer	166	7.8
1 FDR/SDR w/breast cancer	419	19.6
2 FDR/SDR w/breast cancer	729	34.2
3+ FDR/SDR w/breast cancer	820	38.4
FDR w/breast cancer	1377	64.5
FDR/SDR w/ovarian cancer	374	17.5
FDR/SDR w/colon cancer	394	18.5
Breast cancer pathology (*n* = 2425 cancers)		
Behavior		
Invasive	1727	71.2
In situ	205	8.5
Unknown	493	20.3
Histology^c^		
Ductal	1210	49.9
Lobular	164	6.8
Mixed	132	5.4
Other	183	7.5
Unknown	736	30.4
Grade		
Low	232	10.9
Intermediate	581	27.2
High	581	27.2
Unknown	1031	42.5
ER status		
Positive	1203	49.6
Negative	349	14.4
Indeterminate	5	0.2
Unknown	868	35.8
HER2 status		
Negative	888	36.6
Positive	289	11.9
Indeterminate	47	1.9
Unknown	1201	49.5
Breast cancer full HR status known (*n* = 1055)		
ER+Her2−	651	61.7
ER+Her2+	163	15.5
ER−Her2+	93	8.8
ER−Her2−	148	14.0

*FDR* first degree relative, *SDR* second degree relative

^a^ For race, other refers to American Indian, Alaskan Native, Native Hawaiian, Other Pacific Islander, or mixed race

^b^ Age of BC diagnosis was unknown for seven cases

^c^ For histology, other refers to medullary, mucinous, tubular

**Fig. 1:**
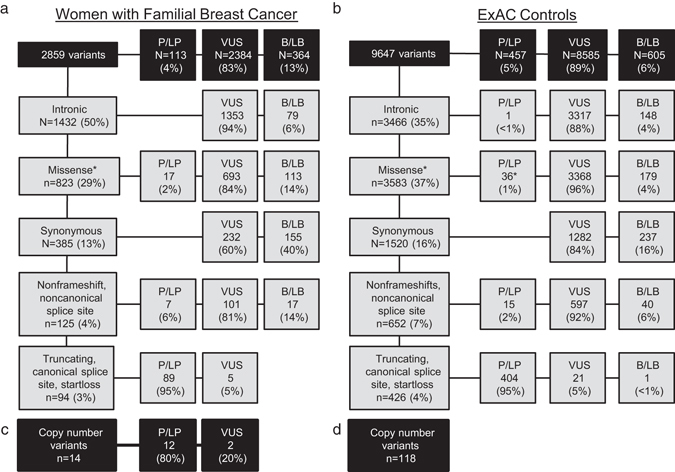
Variant classes identified in familial breast cancer women and Exome Aggregation Consortium (ExAC) controls. All single nucleotide and insertion/deletion variants in the 26 studied genes passing sequencing quality filters and with alternate allele frequencies (AAF) between 30 and 70% were classified by the described variant classification methodology. Variants are divided by general type (noncoding, missense, synonymous, loss of function and other) followed by the breakdown of pathogenic/likely pathogenic (P/LP) variants, variants of uncertain significance (VUS), and benign/likely benign (B/LB) variants in (**a**) familial breast cancer cases and (**b**) ExAC (The Cancer Genome Atlas excluded) non-Finnish European controls. *Asterisks*: Includes *CHEK2* low-risk alleles p.I157T and p.S428F and *MUTYH* heterozygous pathogenic mutations in both data sets. The Exome hidden Markov model and COpy number Detection by EXome sequencing algorithms were used to identify copy number variants (CNVs) in the cases (**c**) and analysis of ExAC genome browser data was analyzed to identify CNVs in the controls (**d**)

From the 2134 women, 2859 heterozygous single nucleotide or insertion/deletion variants were identified in 26 known or proposed breast cancer susceptibility genes (Fig. 1). Variants were classified using a modified American College of Medical Genetics and Genomics variant classification pipeline (Methods).^16^ Variants of uncertain significance (VUS) and likely benign/benign variants accounted for 96% of identified variants. We classified 114 (4%) of the variants as pathogenic or likely pathogenic mutations (further addressed as “mutations” throughout). In addition, analysis of next generation sequencing (NGS) data by a combination of the exome hidden Markov model^17^ and COpy number Detection by EXome sequencing^18^ algorithms, identified 14 germline copy number variants, 12 of which were classified as likely pathogenic (Supplementary Table 1). Therefore, counting single nucleotide variants, indels and copy number variants, 126 unique mutations were identified. As some mutations were recurrent, 183 total mutations were identified in FBC patients. The distribution of mutations by mutation type for each of the 26 analyzed genes are shown in Table 2. The same variant classification pipeline was then applied to the 9647 single nucleotide or insertion/deletion mutations identified in the 26 genes in ExAC data from non-Finnish European individuals (excluding samples from The Cancer Genome Atlas). As was seen in FBC women, 95% of the analyzed variants were classified as VUS or likely benign/benign and 5% of variants were classified as mutations (Fig. 1). Analysis of published exome hidden Markov model data for ExAC revealed 118 copy number variants in these genes.^19^ The majority of mutations identified were truncating variants in both FBC women and ExAC controls (Table 2).

**Table 2:** Breakdown of types of pathogenic and likely pathogenic mutations identified in familial breast cancer women and ExAC controls

	Breast cancer cases^a^						EXAC controls						
	Rates		Mutation type^b^				Rates		Mutation type^b^				
Gene	*n*	% of 2134	*I*/*N*	*S*	*M*	*C*	*n*	% of 26,375	*I*/*N*	*S*	*M*	*C*	Other
# of unique mutations	126						457						
Total # of mutations identified	181						1612						
Total # of mutation carriers^a^	175	8.2					1612	6.1					
# of BC-associated mutation carriers^c^	100	4.7					441	1.7					
High risk breast cancer^a^	30	1.41					57	0.22					
*PALB2* ^a^*	19	0.89	19	0	0	0	33	0.13	28	2	0	2	1
TP53*	11	0.52	2	1	8	0	18	0.07	1	1	15	1	0
*CDH1*	0	0.00	0	0	0	0	4	0.02	2	1	0	1	0
*PTEN*	0	0.00	0	0	0	0	1	0.00	1	0	0	0	0
*STK11*	0	0.00	0	0	0	0	1	0.00	0	0	0	1	0
Moderate risk breast cancer^a^	68	3.19					403	1.53					
*CHEK2* ^a^*	35	1.64	25	2	3	5	257	0.97	156	13	61	27	0
*ATM* ^a^*	32	1.50	19	6	2	5	100	0.38	58	22	5	8	7
*NBN*	1	0.05	1	0	0	0	46	0.17	34	7	0	5	0
Proposed breast cancer^a^	39	1.83					621	2.35					
BARD1*	7	0.33	6	1	0	0	33	0.13	25	2	0	6	0
*FANCM*	7	0.33	7	0	0	0	185	0.70	181	3	0	1	0
*BLM*	6	0.28	4	2	0	0	64	0.24	51	8	2	3	0
*RAD50* ^a^	4	0.19	4	0	0	0	79	0.30	75	4	0	0	0
*RAD51D*	4	0.19	3	1	0	0	7	0.03	6	0	0	1	0
*RAD51C* ^a^	3	0.14	2	0	0	1	35	0.13	26	6	0	3	0
*BRIP1*	2	0.09	2	0	0	0	46	0.17	39	3	0	4	0
*PPM1D*	2	0.09	2	0	0	0	27	0.10	25	0	0	2	0
*FANCC*	1	0.05	1	0	0	0	53	0.20	23	26	0	4	0
*MRE11A*	1	0.05	1	0	0	0	28	0.11	19	4	0	5	0
*RINT1*	1	0.05	1	0	0	0	39	0.15	21	16	0	2	0
*XRCC2*	1	0.05	1	0	0	0	13	0.05	8	2	0	3	0
*BAP1*	0	0.00	0	0	0	0	12	0.05	10	0	0	2	0
Lynch syndrome^a^	11	0.52					134	0.51					
*MSH6* ^a^	6	0.28	6	0	0	0	35	0.13	27	3	2	3	0
*MSH2*	3	0.14	0	1	1	1	8	0.03	3	1	1	3	0
*PMS2*	2	0.09	2	0	0	0	77	0.29	25	6	18	26	2
*MLH1*	0	0.00	0	0	0	0	14	0.05	2	3	5	4	0
*MUTYH* monoallelic	33	1.55	3	4	26	0	397	1.51	21	9	366	1	0

^a^ Double mutation carriers are counted once in the total number of mutation carriers. In the individual gene counts, cases with two pathogenic mutations are counted in the totals for both genes. Mutations are in *CHEK2*(4), *ATM*(3), *PALB2*(2), *RAD50*(2), *MSH6*(1), and *RAD51C*(1)

^b^ Mutation type *I*/*N* indels and nonsense mutations, *S* splicing, *M* missense, *C* copy number variant, *O* nonconsensus splice site or other noncoding mutations

^c^ Counting mutation carriers of genes with statisically significant odds ratios in case–control analyses (*)

**Fig. 2:**
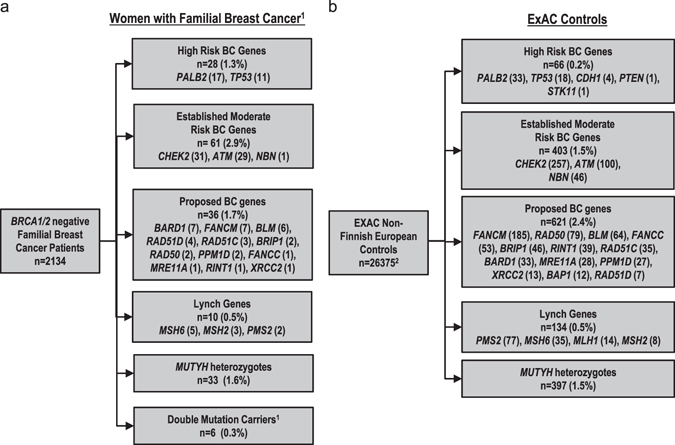
Spectrum of mutations in 26 genes in familial breast cancer women compared to Exome Aggregation Consortium (ExAC) controls. Rates of truncating, known pathogenic missense mutations, and copy number variants in (**a**) *BRCA1/2* negative familial breast cancer women analyzed by targeted sequencing and (**b**) analysis of ExAC data (excluded copy number variation). Sequencing data was analyzed for mutations in high risk breast cancer genes, moderate risk breast cancer genes, proposed breast cancer genes, the Lynch syndrome genes, and *MUTYH*. ^1^Women with two pathogenic mutations were removed from single gene counts and include two cases with *ATM/CHEK2* mutations, two with *CHEK2/PALB2* mutations, and one patient each with *ATM/RAD50*, and *MSH6/RAD50* mutations. ^2^Denominator for ExAC non-Finnish European controls (The Cancer Genome Atlas excluded) was determined by averaging the allele number for each identified variant and dividing by two.

From the FBC women, 183 mutations were identified in 176 women (8.2%), or 6.6% when excluding 33 women with monoallelic *MUTYH* mutation carriers (1.6% of cases) (Fig. 2; Table 2). The majority of women had only a single mutation identified. However, six women (0.3%) were double mutation carriers, with mutations found in two different genes, including two in *ATM*/*CHEK2*, two in *PALB2*/*CHEK2*, and one each of the following: *ATM*/*RAD50* and *MSH6*/*RAD50*. Evaluation of the ExAC data for mutations in the 26 genes used in the study identified 1612 mutation carriers (6.1%), or 4.6% when excluding 397 monoallelic *MUTYH* mutation carriers (1.5% of controls) (Fig. 2; Table 2). Mutations were enriched in known high (odds ratio (OR) > 5) and moderate-risk (OR 2–5) genes among FBC women compared to ExAC controls (1.5 vs. 0.2% and 3.2 vs. 1.5%, respectively, *p* values <0.05) (Table 2). In contrast, the frequency of mutations in proposed breast cancer genes was greater in ExAC controls than among women with FBC (2.4 vs. 1.8%, *p* = 0.03). The proportions of individuals with heterozygous *MUTYH* and Lynch syndrome mutations were nearly identical between FBC women and ExAC controls (~1.5 and 0.5% respectively in both groups, *p* > 0.05).

Mutations in high-risk breast cancer genes or genes associated with syndromic diagnoses (*CDH1, PALB2, PTEN, STK11*, and *TP53* ^6, 20, 21^) were identified in 1.4% of the FBC cohort (Table 2). Excluding carriers of copy number variants and double mutation carriers, comparing women with FBC to ExAC controls, *TP53* and *PALB2* showed significant ORs of 8.17 (95% CI 3.74–18.26, *p* = 2.23 × 10^−6^) and 6.95 (CI 3.71–12.70, *p* = 2.33 × 10^−8^), respectively (Table 3). The average age at breast cancer diagnosis for *TP53* mutation carriers was significantly younger than for mutation-negative individuals (Table 4) (age at diagnosis 39 years vs. 48 years, *p* = 8 × 10^−5^), and *TP53* mutation carriers were more likely to have bilateral breast cancer than mutation-negative individuals (36 vs. 13%, *p* = 0.05). Compared to mutation-negative women, *TP53* mutation carriers were not enriched for a family history of ovarian cancer (9% in mutation carriers vs. 17% in non-carriers, *p* > 0.05) or for first degree relatives with breast cancer (*p* > 0.05). Importantly, only five out of 11 (45%) *TP53* mutation carriers met classic Li Fraumeni syndrome (LFS) or Chompret criteria, or had breast cancer diagnosed under the age of 31.^20^ *TP53* mutation carriers also had a significantly higher proportion of HER2+ breast cancer compared to mutation-negative cases (83 vs. 24% in non-carriers, *p* = 0.004). In contrast, *PALB2* mutation carriers were more likely to have ER−HER2− breast cancer (46% in mutation carriers vs. 14% in non-carriers, *p* = 0.006) and less likely to have ER+HER2− breast cancer (31% in mutation carriers vs. 62% in non-carriers, *p* = 0.04) compared to mutation-negative women. Of note, two women carrying the same *CDH1* VUS, currently discordant in Clinvar as either a VUS or likely pathogenic (c.1118C>T, p.Pro373Leu), had no family history of gastric cancer and therefore did not meet criteria for hereditary diffuse gastric cancer syndrome. Both women had a history of invasive ductal (at ages 34 and 36) rather than lobular carcinoma.

**Table 3:** Case–control analysis

Gene	Case AC^a^	Case AN	Case AF	Control AC	Control AN	Control AF	OR	95% CI	*p*-value
Gene (*p* < 0.05 and case number >5)^b^									
*ATM*	24	4254	0.00564	92	53288	0.00173	3.28	2.06–5.21	**3.64E−06**
*BARD1*	7	4254	0.00165	27	52157	0.00052	3.18	1.34–7.36	**1.22E−02**
*PALB2*	17	4254	0.004	31	53738	0.00058	6.95	3.71–12.70	**2.33E−08**
*TP53*	11	4254	0.00259	17	53579	0.00032	8.17	3.74–18.26	**2.23E−06**
*CHEK2* ^*trunc*^	23	4254	0.00541	169	50448	0.00335	1.617	1.03–2.51	**4.11E−02**
Gene (*p* > 0.05 or case number < 5)									
*RAD51D*	4	4254	0.00094	6	53110	0.00011	8.33	2.20–30.48	**4.41E−03**
*BLM*	6	4254	0.00141	61	53468	0.00114	1.24	0.52–2.85	6.36E−01
*BRIP1*	2	4254	0.00047	42	53681	0.00078	0.60	0.10–2.33	7.70E−01
*FANCC*	1	4254	0.00024	49	52870	0.00093	0.25	0.01–1.48	1.81E−01
*FANCM*	7	4254	0.00165	184	53071	0.00347	0.47	0.22–1.01	5.12E−02
*MRE11A*	1	4254	0.00024	23	53534	0.00043	0.55	0.03–3.22	1.00E+00
*MSH2*	2	4254	0.00047	5	50210	0.0001	4.72	0.67–22.80	9.84E−02
*MSH6*	5	4254	0.00118	32	52301	0.00061	1.92	0.71–4.83	1.97E−01
*MUTYH*	33	4254	0.00776	396	53063	0.00746	1.04	0.72–1.48	7.82E−01
*NBN*	1	4254	0.00024	41	52529	0.00078	0.30	0.02–1.79	3.71E−01
*PMS2*	2	4254	0.00047	51	49235	0.00104	0.45	0.08–1.72	4.40E−01
*PPM1D*	2	4254	0.00047	25	47537	0.00053	0.89	0.15–3.47	1.00E+00
*RAD50*	2	4254	0.00047	79	52897	0.00149	0.31	0.06–1.15	9.14E−02
*RAD51C*	1	4254	0.00024	32	53293	0.0006	0.39	0.02–2.41	5.13E−01
*RINT1*	1	4254	0.00024	37	53662	0.00069	0.34	0.02–2.06	5.25E−01
*XRCC2*	1	4254	0.00024	10	53987	0.00019	1.27	0.06–8.63	5.66E−01

*AC* allele count, *AN* allele number, *AF* allele frequency, *OR* odds ratio, *CI* confidence interval, *CHEK2trunc CHEK2* truncating mutations

^a^ Cases with two mutations and CNVs were excluded from Case AC

^b^ CHEK2 variants in aggregate did not reach statistical significance (See text)

**Table 4:** Cancer history and breast cancer pathology characteristics of mutation carriers compared to non-mutation carriers

	No mutation	Mutation carrier	*ATM*	*CHEK2*	*PALB2*	*TP53*	Candidate breast^a^	Ovarian^a^	Lynch^a^	Double carriers^a^
Total # of cases	1936	143	29	31	17	11	35	9	10	6
Average age of onset	48	45	47	44	46	39	46	44	49	44
		*p* = 8e−5		*p* = 0.02		*p* = 1e−3				
Personal cancer history										
Bilateral breast cancer	252	26	6	7	2	4	2	0	1	4
	13%	18%	21%	23%	12%	36% *p* = 0.05	6%	0%	9%	67% *p* = 0.004
Ovarian cancer	27	5	0	1	1	0	1	1	2	0
	1%	3%	0%	3%	6%	0%	3%	11%	22%	0%
Any other cancer	153	12	3	4	0	1	1	0	2	1
	8%	8%	10%	15%	0%	10%	3%	0%	18%	17%
Family cancer history										
FDR w/Breast cancer	1247	93	18	27	10	7	21	6	7	6
	64%	65%	62%	87% *p* = 0.007	59%	64%	60%	67%	64%	100%
FDR/SDR w/ Ovarian cancer	327	31	4	6	1	1	10	4	3	1
	17%	22%	14%	19%	6%	9%	29%	44%	27%	17%
Any relative w/ Colon cancer	348	34	8	9	2	3	7	3	2	2
	19%	24%	28%	29%	12%	27%	20%	38%	18%	33%
Breast cancer pathology										
Total # w/ER Status	1400	112	25	20	16	6	27	6	8	5
ER+BC	1083	88	22	18	10	3	18	3	8	4
	77%	79%	88%	90%	63%	50%	64%	50%	100%	80%
Total # w/Her2 Status	1063	85	18	12	13	6	26	7	4	5
Her2+BC	259	22	5	5	2	5	3	2	0	0
	24%	26%	28%	42%	15%	83% *p* = 0.004	12%	29%	0%	0%
Total # w/ER +Her2 status	1055	85	18	12	13	6	24	6	4	4
ER+Her2−	651	48	13	7	4	1	15	2	4	3
	62%	56%	72%	58%	31% *p* = 0.04	17%	63%	33%	100%	75%
ER–Her2−	148	15	0	0	6	0	8	3	0	1
	14%	18%	0%	0%	46% *p* = 0.006	0%	33%	50%	0%	20%
ER+Her2+	163	16	4	4	3	2	2	1	0	0
	15%	19%	22%	33%	23%	33%	8%	17%	0%	0%
ER–Her2+	93	6	1	1	0	3	1	0	0	0
	9%	7%	6%	8%	0%	50% *p* = 0.01	4%	0%	0%	0%

^a^ Candidate Breast: candidate breast cancer susceptibility genes (*BARD1, BLM, FANCC, FANCM, MRE11A, PPM1D, RAD50, RINT1, XRCC*2); Ovarian: Ovarian Cancer Genes (*BRIP1, RAD51C, RAD51D*); Lynch: Lynch syndrome genes (*MLH1, MSH2, MSH6, PMS2*); Double carriers: two women with *ATM/CHEK2* mutations, two with *CHEK2/PALB2* mutations, and one woman each with *ATM/RAD50* and *MSH6/RAD50* mutations

The *ATM, CHEK2*, and *NBN* ^6^ moderate-risk breast cancer genes^6, 22^ had the highest mutation rate in the FBC cohort at 3.19%, including five of the double mutation carriers. In the case–control analysis, excluding copy number variants and double mutation carriers, mutations in *ATM* were significantly enriched in FBC women compared to ExAC controls, OR = 3.28, 95% CI 2.06–5.21, *p* = 3.64 × 10^−6^ (Table 3). *CHEK2* truncating mutations (OR = 1.62, 95% CI 1.03–2.51, *p* = 0.041) (Table 3) and *CHEK2*, p.1100delC alone (OR = 1.85, 95% CI 1.13–3, *p* = 0.017) were also enriched in FBC.^23, 24^ The personal history of bilateral breast cancer and other cancers were not statistically significantly higher for *ATM* or *CHEK2* mutation carriers compared to mutation-negative women (Table 4). Cancer family history was also similar among these groups when compared to mutation-negative women, except that *CHEK2* mutation carriers had a higher proportion of first-degree relatives with breast cancer (87% in mutation carriers vs. 64% in non-carriers, *p* = 0.007). None of the breast cancers arising in *ATM* and *CHEK2* mutation carriers, for which hormonal status was available, were histologically classified as ER−HER2–, consistent with previous reports^25^ (Table 4). Only one case was identified with the common Slavic pathogenic variant *NBN* c.657del5^6^ (Fig. 2); therefore precluding a case–control analysis.

Mutations in candidate breast cancer genes were only observed in 1.7% of women in this cohort (Fig. 2). Of these, only *FANCM, BLM*, and *BARD1* had mutations in over five FBC women. Only *BARD1* mutations were significantly associated with an increased risk of breast cancer (OR = 3.18, 95% CI 1.34–7.36, *p* = 0.012) in case–control analysis. The rates of bilateral breast cancer, ovarian cancer, family history of breast cancer in a first degree relative, family history of ovarian cancer, or pathological characteristics of the breast cancer were not significantly different in these women compared to mutation-negative women (Table 4). In addition, there were nine women with mutations in recently described ovarian cancer susceptibility genes, *BRIP1*, *RAD51C*, and *RAD51D*.^22^ A personal history of ovarian cancer only was reported for only one case; furthermore, these mutation carriers were not statistically more likely to have a first-degree or second-degree relative with ovarian cancer compared to mutation-negative individuals (Table 4).

Mutations in genes classically predisposing to colorectal cancer were infrequently observed. Mutations in Lynch syndrome (LS) genes (*MLH1, MSH2, PMS2, MSH6*) were identified in 11 cases, including one case with mutations in both *MSH6* and *RAD50*. Six *MSH6* mutations were detected among FBC cases, but this rate was not significantly higher than in ExAC control individuals (0.28 vs. 0.13%). Mutations in other LS genes also were not significantly associated with FBC. The age of onset of breast cancer in women with LS mutations and mutation-negative individuals was similar (age of onset 49 vs. 48, *p*-value not significant), and there were no differences in the rates of bilateral breast cancer or family history of breast or ovarian cancer (Table 4) or colon cancer (data not shown). Three of the ten LS gene mutation carriers had a personal history of ovarian or endometrial cancer. However, none had a personal history of colon cancer and only two had a family history of colon cancer (data not shown). All breast cancers for which information was available were classified as ER+. There were no phenotypic features identified specific to *MUTYH* mutation carriers; specifically, the rate of colon cancer family history was not higher in *MUTYH* mutation carriers compared to mutation-negative individuals (data not shown). No mutations were identified in *BAP1*, *MLH1*, *PTEN*, or *STK11*.

The frequency of copy number variants in genes associated with breast cancer in women with FBC has been largely understudied. Using COpy number Detection by EXome sequencing^18^ and the exome hidden Markov model,^17^ we identified copy number variants in 14 cases (0.7%), and classified 12 as mutations (Table 2, Supplementary Table 1). Two copy number variants, a whole gene duplication of *STK11* and a single exon in-frame deletion in *PTEN* were classified as VUS. Five individuals had copy number variants in *CHEK2*, five in *ATM*, one in *RAD51C* and one in *MSH2*. Copy number variants in *ATM*, *CHEK2*, and *RAD51C* were confirmed by multiplex ligation-dependent probe amplification (MLPA) in the 11 individuals for which DNA was available (Supplementary Fig. 1). Interestingly, the *MSH2* deletion was from an individual meeting Amsterdam I/II criteria for LS testing, and the individual with a *RAD51C* deletion had a personal history of both breast and ovarian cancer. As there were five or more individuals with copy number variants in *ATM* and *CHEK2* each, a case–control analysis was performed individually for copy number variants identified in these genes in the FBC cohort compared to copy number variants identified in the ExAC cohort. Copy number variants in *CHEK2* (OR = 3.2, CI 95% 1.64–5.85, *p* = 4.5 × 10^−6^) and *ATM* (OR = 7.7, 95% CI 3.13–18.1, *p* = 9.64 × 10^−6^) were enriched in the FBC women.

## Discussion

We have described the spectrum of mutations in known or proposed breast cancer susceptibility genes in a large cohort of 2134 FBC patients. Overall, 8.2% of patients were found to carry a mutation in the genes analyzed. Mutations in *ATM*, *CHEK2*, and *PALB2* accounted for the majority of mutations (4%), consistent with other studies showing that these are the most commonly mutated genes other than *BRCA1* and *BRCA2* that are associated with an increased risk of FBC.^9, 10, 14, 26^ While the assayed genes differ slightly between previously reported studies, our results showing a 4.3% mutation rate (Fig. 2) in high and moderate risk breast cancer predisposition genes among *BRCA*-negative FBC women, are also consistent with these reports.

In the present FBC cohort, only 1.3% of women carried a mutation in a high-risk breast cancer gene (Fig. 2). This risk estimate for *TP53* mutations (OR = 8.17, 95% CI 3.74–18.26) has substantially tighter confidence intervals than previously reported risk estimates (OR = 11, 95% CI 0.6−201).^14^ However, further studies providing functional and risk associated annotation of variants, and accounting for contamination of true germline *TP53* mutations with mosaic mutations that arise due to clonal hematopoiesis,^27^ are needed to define accurate risk estimates for breast cancer associated with *TP53* inactivating mutations. *PALB2* mutations were also associated with a high-risk of breast cancer (OR = 6.95, 95% CI 3.71–12.70), consistent with prior data.^14^ Our results support the rarity of *CDH1*, *PTEN*, and *STK11* mutations in FBC cohorts and the classification of *TP53* and *PALB2* as high risk genes (OR > 5) for FBC. Adding samples with unexpected *BRCA1/2* findings to the cohort of samples passing quality filters (*n* = 2211), it is important to point out that more *BRCA1/2* mutations were incidentally identified in a cohort of reportedly *BRCA1/2* negative individuals (3.5%) than mutations in the other high risk genes combined (1.3%). These results confirm that mutations in high risk genes outside of *BRCA1/2* are exceedingly rare in FBC patients.

Regarding published moderate-risk genes (OR 2–5), this is the first large multigene case–control study that was able to estimate risks of FBC associated with mutations in *ATM* (OR = 3.28, 95% CI 2.06–5.21). *CHEK2* truncating mutations, and c.1100delC alone were also associated with increased risks of breast cancer; however below an OR of 2, thus classifying *CHEK2* as a low risk gene for FBC. Larger studies will be needed to accurately define the risk of breast cancer associated with missense mutations in these genes.

In the proposed breast cancer gene group, mutations in *BARD1* were found significantly more frequently in individuals with FBC than in ExAC controls (OR = 3.18, 95% CI 1.34–7.36, *p* = 0.012) (Table 3). This is the first large study to show that *BARD1* is a moderate risk breast cancer predisposition gene for FBC. Similarly, mutations in *RAD51D* were associated with increased risks of breast cancer (OR = 8.33, 95% CI 2.2−30.48, *p* = 0.0044). However, these findings should be interpreted with caution because the risks were based on four mutation carriers and are likely unstable. Mutation rates overall in LS genes were almost identical in FBC women and ExAC controls. However, mutations in *MSH6* and *MSH2* were substantially enriched (Table 2) and merit further consideration as FBC predisposition genes.^21^ Heterozygous mutations in *MUTYH* were not associated with increased breast cancer risk. Mutations in the proposed breast cancer gene groups (*FANCM*, *BLM*, *RAD50*, *RAD51C*, *BRIP1*, *PPM1D*, *FANCC*, *MRE11A*, *RINT1*, *XRCC2*, and *BAP1*) were rare. Therefore, the contribution of these genes to breast cancer risk could not be assessed.

Our results showing a significant association of FBC with mutations in *ATM*, *BARD1*, *CHEK2*, *PALB2* validate a recent study investigating the association of breast cancer in a cohort of individuals who underwent genetic testing at a large testing company.^28^ The results here, albeit on a much smaller sample size, are derived from a cohort with well-curated family and personal cancer histories (instead of information only obtained from clinical testing report forms) and therefore provide needed validation of the results from a broad sample of individuals for whom less clinical information is available.

Consistent with other reports regarding genotype–phenotype correlations (Table 4), *TP53* mutation carriers had a significantly higher portion of HER2+ breast cancer than those without mutations.^29^ Forty-five percent of *TP53* mutation-positive women met LFS criteria. Since many women and families included in the current study were ascertained prior to multigene panel testing, emergence of well-defined medical management recommendations for LFS, and recent revisions in LFS criteria, it is likely that many of these cases would have been offered genetic testing under current standards of clinical care.^4^ *PALB2* mutation carriers were more likely to have ER−HER2− breast cancer, whereas *ATM* and *CHEK2* mutations were never associated with ER−HER2− breast tumors.^25^ *BRIP1*, *RAD51C*, and *RAD51D* mutation carriers were not more likely to have a first-degree or second-degree relative with ovarian cancer compared to mutation-negative FBC women, inconsistent with prior studies associating mutations in these genes with high-risks of ovarian cancer.^22^

This study is one of the first to thoroughly evaluate breast cancer predisposition genes included in typical multigene panels in *BRCA*-negative FBC and to estimate risks of cancer by comparison with the ExAC reference control dataset. Assuming one predisposition gene mutation on average per ExAC control individual, approximately 1.7% of controls had a high or moderate-risk breast cancer gene mutation and 4.4% had mutations in the 26 genes under evaluation (Fig. 2). The high mutation carrier frequency adds to previous data from control populations and clarifies the likelihood of finding unexpected or incidental hereditary cancer predisposition gene mutation carriers in the unaffected population.^14^ This also strengthens the justification for counseling about the potentially broad spectrum of genetic findings for individuals undergoing genetic risk assessment.^2, 30^ However, these results should be evaluated with caution as different ascertainment and sequencing techniques were used in the ExAC controls compared to the FBC cohort. In addition, the ethnic makeup of the FBC cohort and ExAC controls likely differs and it is possible that ORs may be over or underestimated based on differences in ethnic makeup or number of individuals of Ashkenazi Jewish status. Finally, the associations of FBC with *BARD1* and *CHEK2* truncating mutations are no longer significant when accounting for multiple testing (*p*-value threshold of <0.002), and therefore require validation in larger case–control studies.

Twelve individuals with breast cancer were identified as having copy number variants classified as likely pathogenic in these genes. Copy number variant analysis using NGS is promising, but is still in development. This is the first case–control analysis of this kind to include copy number variant evaluation. Copy number variants in *CHEK2* and *ATM* showed significant associations. Of note, *ATM* showed a significantly elevated OR of 7.7 (95% CI 3.13–18.1, *p* = 9.64 × 10^−6^). Copy number variants were particularly enriched in the FBC women and will require further study. It is possible the FBC women with identified copy number variants represents an underestimate of the total number of copy number variant findings. However, it is unlikely that unidentified copy number variants account for the majority of the genetic etiology in mutation-negative FBC women. Additional research will be needed on this topic given the increasing use of NGS copy number variant calling for copy number variant detection by commercial genetic testing laboratories.

If we consider only mutations in genes that reached statistical significance in our analysis, (*TP53*, *ATM*, *PALB2*, *BARD1*, and truncating mutations in *CHEK2*), approximately 4.7% of *BRCA* negative FBC women have mutations in breast cancer susceptibility genes. Given that *BRCA* mutations are responsible for another 5–10% of attributable risk,^7, 31^ our results suggest that 86–91% of FBC women have no underlying genetic etiology that can be identified by mutational analysis of the exonic regions of genes on the majority of current breast cancer multigene panels, supporting the concept of missing heritability in breast cancer.^14, 26^ In this study over 90% of variants identified in these categories among our 26 selected genes were determined to be VUS (Fig. 1). Thus, additional research is needed to study the contribution of intronic, missense, and synonymous variants in the current panel genes and other candidate genes to FBC.

As shown above, the risk estimates for mutations in many proposed breast cancer genes did not reach statistical significance. Based on these data, clinical actionability of genes with low or undefined-risk remains unclear. Results from this study support targeting the known clinically-actionable high and moderate-risk genes with multigene panels as a first-tier approach to understanding breast cancer susceptibility.

## Methods

### The simplexo targeted resequencing

#### Case and control selection

2266 *BRCA*-negative women with FBC defined as a proband with breast cancer and at least two first-degree to third-degree relatives with breast or ovarian cancers under age 70 years were selected from multiple centers (City of Hope, University of Pennsylvania, Mayo Clinic, Memorial Sloan Kettering, British Columbia Cancer Agency, the FIRC Institute of Molecular Oncology, European Institute of Oncology, Stanford, Dana Farber Cancer Institute, and the National Institutes of Health) and their germline DNA (from blood or saliva) was sequenced for 26 known or proposed breast cancer susceptibility genes at Mayo Clinic and at the University of Pennsylvania. Germline DNA from two-hundred non-cancer controls from one of the contributing centers was used to estimate systematic sequencing artifacts of NGS. All individuals were consented and enrolled into the study through center specific Institutional Review Board approved protocols. Sequencing was completed once per sample.

#### Target selection and library preparation

The target region for sequencing of the candidate breast cancer susceptibility genes were selected based on coding exons. For established genes, intragenic regions were also included. Baits for solution based hybrid selection capture^32^ were designed using the Agilent SureSelect design tool (https://earray.chem.agilent.com/suredesign/). 500 nanograms of high molecular weight DNA was fragmented using a Covaris E-220. Fragmentation was verified by bioanalyzer. Library preparation was performed using the NEBNext Ultra DNA Library Prep kit (E7370L), and NEB Dual indexed adapters. Fragmented DNA (mean fragment size 300 ± 20 bp) was end repaired, adapter ligated, and size selected using Ampure XP /SPRI (A63881) and was PCR amplified for eight cycles. A post-PCR clean-up was performed and enrichment for targets was performed using the Agilent SureSelect protocol. Yields were assessed using BioAnalyzer. The mean library size was 300 ± 10 bp. NGS was performed on an Illumina HiSeq 2000 to an estimated 100× mean coverage for the target region to yield paired-end reads of 100 bp per sample, using 24 samples/lane.

#### Computational methods

FASTQ files were de-multiplexed to individual forward and reverse files. Initial QC was performed using the FASTQC^33^ tool kit. Read alignment was performed using BWA^34^ and statistics were generated using bamstats.^35^ The human reference GRCh37 with the addition of decoy regions V5 for improving alignment accuracy and speed was used in all cases.^36^ Sixty-one samples with low quality were removed using the specific criteria of: (1) samples with <1 M reads, (2) low mean bait coverage (<20) over target bases, (3) ±3 S.D. mean heterozygosity, or (4) first-degree relationship from calculated kinship coefficient values (>0.177). Similarly a variant was dropped if the site-specific call rate was less than 50%. Variant calling was performed using the Genome Analysis Toolkit (GATK) v2.6–4. For each sample, the BAM was sorted, duplicates were marked for deletion using Picard,^37^ indels were realigned and base recalibrated. A gVCF was created per sample using the HaplotypeCaller in GATK and all samples were recalled using the Genotype GVCF module. Variants were annotated using SnpEFF, CAVA, and ANNOVAR.^38^

#### Sample and variant level QC/QA

Pre-established criteria were used to identify ineligible individuals. Expected vs. Observed homozygosity using single nucleotide polymorphisms was computed using Plink^39^ and VCFtools^40^ for all samples to identify individual samples from males for removal (Supplementary Figure 2). Similarly, relatedness and ethnicity were estimated using kinship coefficients for all pairwise relationships using identity by state metrics. Individuals with a first-degree relative in the study or suspected duplicate samples were removed. Finally, 77 cases were found to carry *BRCA* mutations (including copy number variants) and were removed. A total of 2134 samples were available for subsequent analyses (Supplementary Figure 1).

In the FBC women sequenced, allelic ratios between 20 and 80% were required for variant inclusion. Allele ratios were not available for ExAC controls. Variants with allele frequencies (AF) > 0.03 in ExAC, ESP6500 and 1000 genomes were excluded (except for the known common pathogenic variant *CHEK2* c.1100delC). The gene set was restricted to genes known (*ATM*, *CDH1*, *CHEK2*, *NBN*, *PALB2*, *PTEN*, *STK11*, and *TP53*) or presumed (otherwise in the list below) to have clinically relevant mutations associated with germline breast cancer susceptibility.^20^ The list included evaluation of introns and exons for *ATM*, *BARD1*, *BLM*, *BRIP1*, *CDH1*, *CHEK2*, *MLH1*, *MRE11A*, *MSH2*, *MSH6*, *MUTYH*, *NBN*, *PALB2*, *PMS2*, *PTEN*, *RAD50*, *RAD51C*, *RAD51D*, *STK11*, *TP53*, and *XRCC2*. Exon-only analysis was available for *BAP1*, *FANCC*, *FANCM*, *PPM1D*, and *RINT1*.

### Variant classification methodology

Variants were classified by two independent methodologies and then subjected to an expert review process for final classification. Researchers classifying the variants were blinded as to the phenotype of the sample from which the variant was derived. In the first methodology, all variants with AF > 0.003 were annotated as VUS, likely benign, or benign. Variants were called pathogenic/likely pathogenic mutations when meeting one of two criteria: (1) Loss-of-function (nonsense, frameshift, splicing +/−1 or 2, whole gene deletion, truncating copy number variants (unless after a known truncating benign variant) and (2) Missense, splicing +/−3+ position, intronic or synonymous variants that were classified as pathogenic or likely pathogenic in ClinVar^41^ by two or more of the following clinical genetics group: Ambry, Sharing Clinical Reports Project, InVitae, GeneDX, Emory, or InSiGHT. One exception to this rule was for *TP53* missense variants, due to the rarity of these variants, where only one group was required. In the second methodology of variant classification, variants were subjected to Ingenuity Variant Analysis (Qiagen Inc, Alameda, CA). The American College of Medical Genetics and Genomics and the Association for Molecular Pathology standards and guidelines for the interpretation of sequence variants were applied to obtain pathogenic/likely pathogenic/VUS/likely benign/benign calls.^42^ In the expert review, results were compared and all concordant calls accepted as final classifications based on the above guidelines.^42^ Discrepancies were evaluated based on literature and database sources and discussed by the variant classification team for consensus on a mutation or VUS/likely benign/benign final classification. Known low-risk alleles were classified in a separate low-risk allele group.

### Case–control analysis

Allele counts for mutations were summed for each gene in FBC cases and non-Finnish European ExAC controls excluding samples from The Cancer Genome Atlas (TCGA) (ExAC v0.3.1 (non-TCGA), http://exac.broadinstitute.org/, accessed 29 December 2014). The ExAC dataset has been used for identifying genes associated with disease and estimating risks.^15, 19, 43, 44^ The allele number for ExAC controls was calculated by averaging the number of the highest quality allele calls across exonic regions. Associations with breast cancer for each gene were generated using a two-sided Fisher’s exact test using minimum likelihood method.

### Identification of copy number variants

Potential copy number variants (one or greater exonic deletions and duplications) were identified with the exome hidden Markov model^17^ and COpy number Detection by EXome sequencing^18^ algorithms from NGS data. Copy number variants identified by both algorithms (*n* = 12) were examined in the Integrated Genome Viewer to confirm the visual presence of a low read count area spanning at least one exon. Copy number variants called were used as independent confirmation of exon deletions/duplications. Separate DNA aliquots than those used for sequencing was available from 12 of the 14 individuals for analysis by MLPA (Supplementary Table 1; Supplementary Figure 2). The ExAC genome browser (http://exac.broadinstitute.org/) data were downloaded and analyzed in the UCSC genome browser (http://genome.ucsc.edu/) for identification of copy number variants.^19^ All gene deletion and duplications spanning large genomic regions beyond a single gene were excluded as were deletions and duplications in repetitive regions, regions corresponding to pseudogenes, and spanning only single exons.

### Clinical and statistical analysis

Clinical data was obtained for the patients in the local tumor set by IRB approved chart review after informed consent. Comparisons of rates in different groups were determined using a two-sided Fisher’s exact test of significance. Using a sample set of 2134 FBC individuals vs. 26,375 controls, this study had >80% power to detect a significant difference in breast cancer risk for genes with a >0.1% control carrier rate leading to relative risk of >4 and for genes with a >0.6% control carrier rate leading to relative risk of >2.

### Data sharing statement

The mutational datasets generated during and/and/or analyzed during the current study are available from the corresponding author on reasonable request.

## Electronic supplementary material

Supplementary Table 1

Supplementary Figure 2

Supplementary Figure 1
