# Author Correction: The contribution of pathogenic variants in breast cancer susceptibility genes to familial breast cancer risk

**DOI:** 10.1038/s41523-017-0046-2

**Published:** 2017-11-07

**Authors:** Thomas P. Slavin, Kara N. Maxwell, Jenna Lilyquist, Joseph Vijai, Susan L. Neuhausen, Steve N. Hart, Vignesh Ravichandran, Tinu Thomas, Ann Maria, Danylo Villano, Kasmintan A. Schrader, Raymond Moore, Chunling Hu, Bradley Wubbenhorst, Brandon M. Wenz, Kurt D’Andrea, Mark E. Robson, Paolo Peterlongo, Bernardo Bonanni, James M. Ford, Judy E. Garber, Susan M. Domchek, Csilla Szabo, Kenneth Offit, Katherine L. Nathanson, Jeffrey N. Weitzel, Fergus J. Couch

**Affiliations:** 1 Department of Medical Oncology, Division of Clinical Cancer Genetics, City of Hope, Duarte, CA USA; 2 Department of Population Sciences, Beckman Research Institute of City of Hope, Duarte, CA USA; 3 Department of Medicine, Division of Hematology-Oncology, Perelman School of Medicine University of Pennsylvania, Philadelphia, PA USA; 4 Abramson Cancer Center, Perelman School of Medicine at the University of Pennsylvania, Philadelphia, PA USA; 5 Department of Health Sciences Research, Mayo Clinic, Rochester, MN USA; 6 Department of Laboratory Medicine and Pathology, Mayo Clinic, Rochester, MN USA; 7 Clinical Genetics Research Lab, Department of Medicine and Department of Cancer Biology and Genetics, Memorial Sloan Kettering Cancer Center, New York, NY USA; 8 Department of Molecular Oncology, British Columbia Cancer Agency, Vancouver, BC Canada; 9 Department of Medical Genetics, British Columbia Cancer Agency, Vancouver, BC Canada; 10 Department of Medicine, Division of Translational Medicine and Genetics, Perelman School of Medicine at the University of Pennsylvania, Philadelphia, PA USA; 11 Clinical Genetics Service, Department of Medicine, Memorial Sloan Kettering Cancer Center, New York, NY USA; 12 IFOM, the FIRC Institute of Molecular Oncology, Milan, Italy; 13 Division of Cancer Prevention and Genetics, European Institute of Oncology, Milan, Italy; 14 Division of Oncology, Stanford University School of Medicine, Stanford, CA USA; 15 Center for Cancer Genetics and Prevention, Dana Farber Cancer Institute, Boston, MA USA; 16 National Institutes of Health, Bethesda, MD USA

**Correction to**: *npj Breast Cancer* (2017); doi:10.1038/s41523-017-0024-8; Published 9 June 2017

Jeffrey N. Weitzel was mistakenly omitted from the statement of co-senior authors, which should have read: “Kenneth Offit, Katherine L. Nathanson, Fergus J. Couch and Jeffrey N. Weitzel jointly supervised this work.”

