# Supplementary material for: The contribution of pathogenic variants in breast cancer susceptibility genes to familial breast cancer risk
Source: NPJ Breast Cancer. 2017 Jun 9;3:22. doi: 10.1038/s41523-017-0024-8 (PMC5466608; doi:10.1038/s41523-017-0024-8)
Supplement: Supplementary file 2 — Supplementary Figure 2 [file 41523_2017_24_MOESM2_ESM.pptx]

## Slide 1
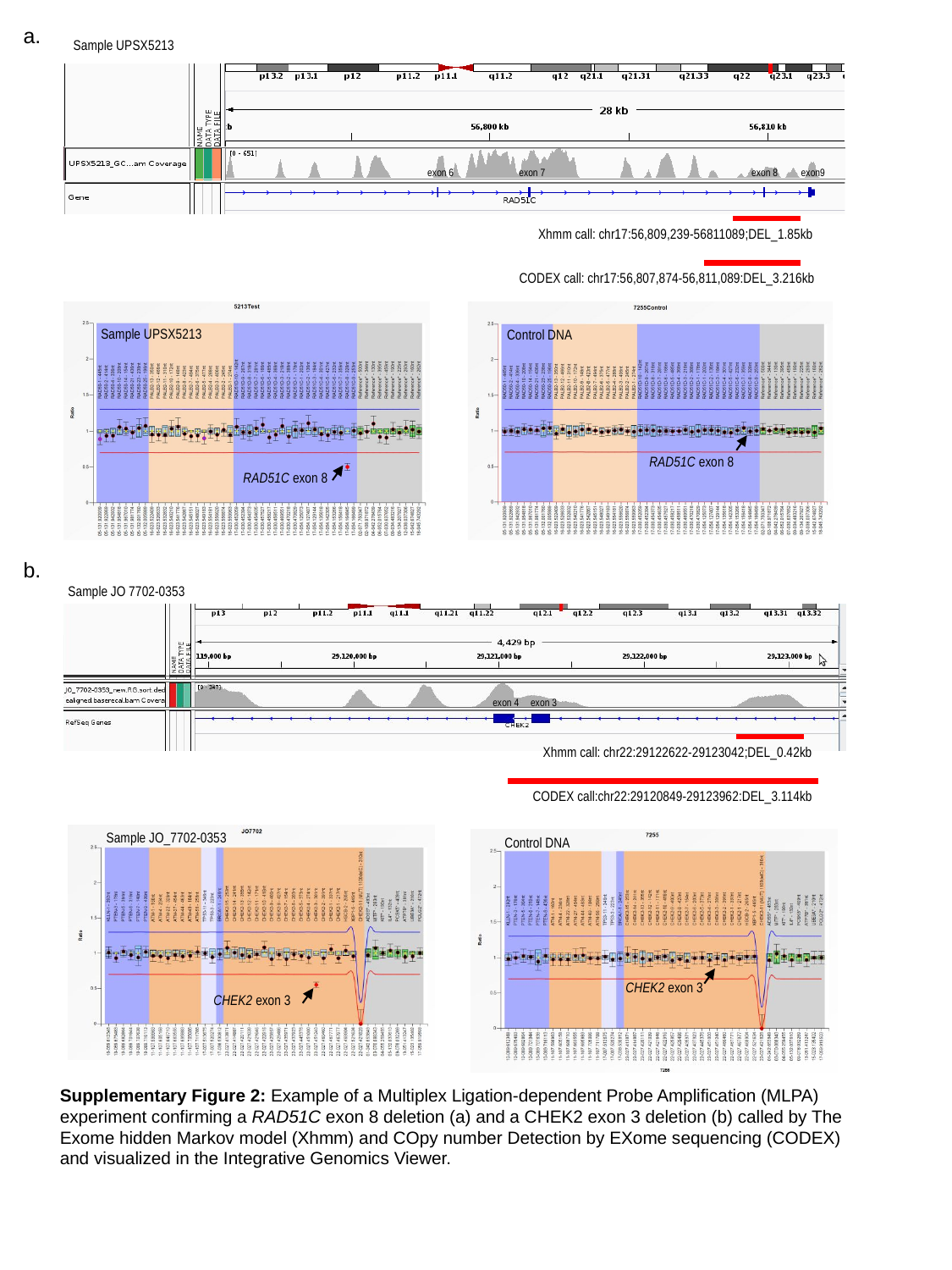

a.
Sample UPSX5213
exon 6 exon 7
exon 8 exon9
Xhmm call: chr17:56,809,239-56811089;DEL_1.85kb
CODEX call: chr17:56,807,874-56,811,089:DEL_3.216kb
Sample UPSX5213
Control DNA
RAD51C exon 8
RAD51C exon 8
b.
Sample JO 7702-0353
exon 4 exon 3
Xhmm call: chr22:29122622-29123042;DEL_0.42kb
CODEX call:chr22:29120849-29123962:DEL_3.114kb
Sample JO_7702-0353
Control DNA
CHEK2 exon 3
CHEK2 exon 3
Supplementary Figure 2: Example of a Multiplex Ligation-dependent Probe Amplification (MLPA) experiment confirming a RAD51C exon 8 deletion (a) and a CHEK2 exon 3 deletion (b) called by The Exome hidden Markov model (Xhmm) and COpy number Detection by EXome sequencing (CODEX) and visualized in the Integrative Genomics Viewer.
