# Supplementary material for: The contribution of pathogenic variants in breast cancer susceptibility genes to familial breast cancer risk
Source: NPJ Breast Cancer. 2017 Jun 9;3:22. doi: 10.1038/s41523-017-0024-8 (PMC5466608; doi:10.1038/s41523-017-0024-8)
Supplement: Supplementary file 3 — Supplementary Figure 1 [file 41523_2017_24_MOESM3_ESM.pptx]

## Slide 1
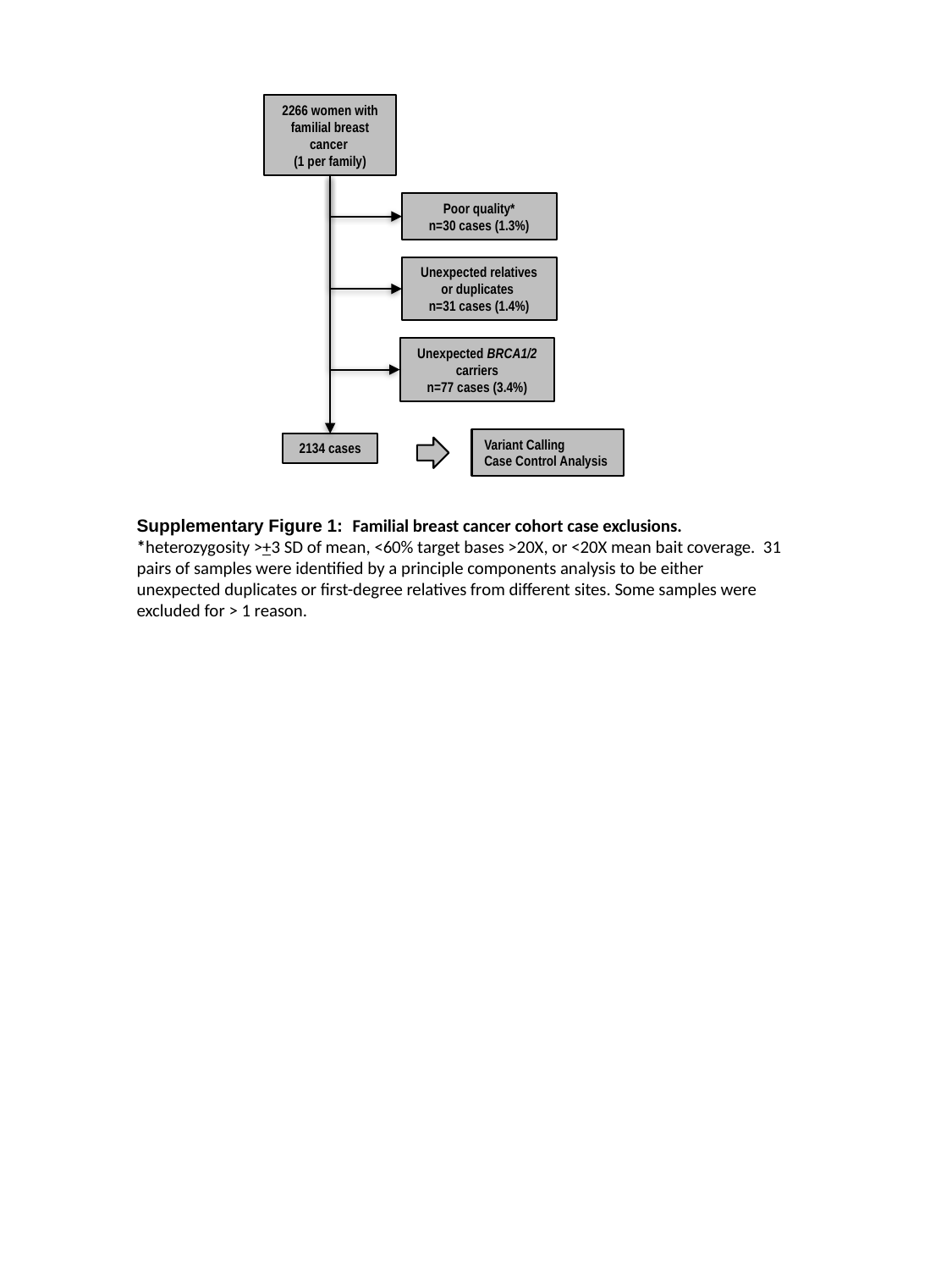

2266 women with familial breast cancer
(1 per family)
Poor quality*
n=30 cases (1.3%)
Unexpected relatives or duplicates
n=31 cases (1.4%)
Unexpected BRCA1/2 carriers
n=77 cases (3.4%)
Variant Calling
Case Control Analysis
2134 cases
Supplementary Figure 1: Familial breast cancer cohort case exclusions. *heterozygosity >+3 SD of mean, <60% target bases >20X, or <20X mean bait coverage. 31 pairs of samples were identified by a principle components analysis to be either unexpected duplicates or first-degree relatives from different sites. Some samples were excluded for > 1 reason.
